# National Medical Union

**Published:** 1914-02-14

**Authors:** Robert Carswell

**Affiliations:** 346 Strand


### THE EDITOR'S LETTER-BOX.

### National Medical Union.

*To the Editor of* The Hospital.

Sir,—I am gratified that our notes in your issue of January 31 should have given Dr. Raiment so much pleasure as to be mistaken by him for an article by the Editor of The Hospital. The analogy between the National Medical Union and the Leipzig Verband, to which it was desired to draw attention, lies in the fact that each originated in a secession from an older society—the Aerztevereinsbund in Germany and the British Medical Association in this country—the inference being that the older Associations were found insufficient to deal with the pressure brought to bear upon the medical profession in both countries by their National Insurance laws. My statement that the origin of the Leipzig Union in 1900 corresponded very closely with that of our National Medical Union was sufficiently amplified in the sentences which followed it, so that if Dr. Raiment's thoughts had not been preoccupied with the idea of trade unionism it would have been quite unnecessary to have requested an explanation.

Dr. Prowse has evidently under-estimated the number of those present at the meeting of the National Medical Guild in Manchester. The authoritative figure—sixty-three—differs by a very small fraction from the thirty or forty of Dr. Prowse, when it is recalled that at the Hammersmith meeting the announcement was made by Dr. Raiment that next month he would be calling a meeting in Manchester of over 2,000.—I am, Sir, yours faithfully,

Robert Carswell.

346 Strand, February 9, 1914.

